# Oxytocin facilitates human touch-induced play behavior in rats

**DOI:** 10.1016/j.cub.2025.05.034

**Published:** 2025-06-23

**Authors:** Himeka Hayashi, Sayaka Tateishi, Ayumu Inutsuka, Sho Maejima, Daisuke Hagiwara, Yasuo Sakuma, Tatsushi Onaka, Valery Grinevich, Hirotaka Sakamoto

**Affiliations:** 1 Department of Biology, Faculty of Environmental, Life, Natural Science and Technology, Okayama University, 3-1-1 Tsushimanaka, Kita-ku, Okayama 700-8530, Japan; 2 Ushimado Marine Institute (UMI), Faculty of Environmental, Life, Natural Science and Technology, Okayama University, Ushimado, Setouchi, Okayama 701-4303, Japan; 3 Division of Brain and Neurophysiology, Department of Physiology, Jichi Medical University, Shimotsuke, Tochigi 329-0498, Japan; 4 Department of Neuropeptide Research in Psychiatry, Central Institute of Mental Health, German Center for Psychiatry (DZPG), Medical Faculty Mannheim, University of Heidelberg, 69120 Mannheim, Germany; 5 Department of Anatomy and Neurobiology, Graduate School of Medical Sciences, Nippon Medical School, 25-16 Nezu 1 Chome, Tokyo 113-8602, Japan

**Keywords:** tickling, oxytocin, oxytocin receptor, ventrolateral part of the ventromedial hypothalamus, affinity-like behaviors

## Abstract

Pleasant touch sensations play a fundamental role in social bonding, yet the neural mechanisms underlying affinity-like behaviors remain poorly understood. Here, we demonstrate that juvenile-adolescent rats, which naturally engage in social play with peers characterized by rough-and-tumble interactions and 50 kHz ultrasonic vocalizations indicating pleasant sensations, develop a strong affinity for human hands through similar playful contact achieved by repeated tickling with human hands. Using this rat with tickling-induced high affinity for human hands, we discovered that repeated tickling mimicking rough-and-tumble play led to increased oxytocin receptor (OTR) expression in the ventrolateral part of the ventromedial hypothalamus (VMHvl). Inhibition of oxytocin signaling in the VMHvl reduced affinity-like behaviors from rats to human hands. These findings suggest that OTR neurons in VMHvl play an important role in the increase in affinity for human hands induced by pleasant touch sensation with human touch-induced play behavior. Based on retrograde and anterograde tracing studies examining the supraoptic nucleus (SON) and the paraventricular nucleus (PVN) as primary sources of oxytocin, we demonstrate that a subset of oxytocin fibers in the VMHvl originate from the SON, suggesting that affinity-like behavior from rats to human hands may be controlled by oxytocin signaling from magnocellular neurons. Together, this work advances our understanding of how oxytocin shapes social behavior and may inform the development of therapeutic strategies to promote positive social interactions.

## Highlights

• Rats develop an affinity for human hands through repeated tickling-like social play
• Human touch-induced play increases OTR expression in the VMHvl
• OTR neurons in the VMHvl are involved in affinity-like behavior to human hands
• There are oxytocin fibers originating from the supraoptic nucleus in the VMHvl

## Graphical abstract

**Figure:**
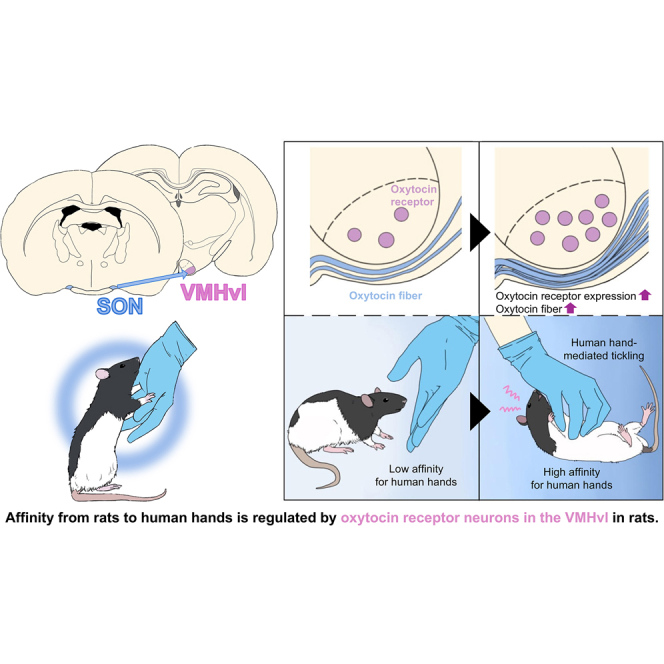

## Teaser

Hayashi et al. demonstrate that human touch-induced pleasurable tickling promotes affinity between rats and human hands. This is accompanied by increased OTR expression in the VMHvl, and blocking the oxytocin signaling inhibits such affinity. Magnocellular oxytocin neurons in the supraoptic nucleus may release oxytocin in the VMHvl.

## Introduction

Social bonding and affinity-like behaviors are fundamental aspects of mammalian behavior. In the natural environment, numerous species exhibit social grouping behaviors despite their capability for solitary survival. This tendency is particularly pronounced in mammals, where offspring survival depends on extended parental care due to their altricial nature at birth. The development of affinity—defined as an enduring emotional bond between specific individuals—serves as a crucial mechanism for maintaining the infant-caregiver relationship and is considered fundamental to the evolution of sociality.

Pleasant tactile stimulation has been identified as a primary facilitator of affinity-like behaviors, as demonstrated by Harlow’s studies with infant rhesus macaques.^1^ In these investigations, infant macaque monkeys presented with two inanimate surrogate mothers—one constructed of wire mesh providing nutrition (“wire-cold mother”) and another covered in soft cloth offering no sustenance (“cloth-warm mother”)—demonstrated a strong preference for the cloth-warm surrogate, only approaching the wire-cold surrogate for feeding purposes.

Juvenile-adolescent rat individuals engage in rough-and-tumble play with their peers, during which they emit 50 kHz ultrasonic vocalizations (USVs) indicating a positive emotional state, pleasure.^2^^,^^3^^,^^4^ Tickling by human hands resembles the type of bodily contact rats obtain during rough-and-tumble play and increases affinity for human hands.^4^^,^^5^^,^^6^ It is thought that tactile stimuli, such as tickling, are received as pleasant touch sensations by rats because they emit 50 kHz USVs, indicating positive emotions, during tickling. Although pleasant touch sensations such as tickling and the hormone oxytocin are both known to be critical cues for affinity-like behaviors,^7^^,^^8^ it remains unclear how these elements are interconnected.

Oxytocin is a nonapeptide hormone produced in hypothalamic regions of the diencephalon, primarily the paraventricular nucleus (PVN) and supraoptic nucleus (SON). Oxytocin plays similarly important roles in social behaviors across both sexes.^9^^,^^10^ Although traditionally recognized for its functions in female reproductive physiology and behaviors,^11^^,^^12^ oxytocin is also crucial for male physiology, as evidenced by increased blood levels after ejaculation, demonstrating the role in male sexual function.^13^^,^^14^^,^^15^ Oxytocin, largely produced by magnocellular neurons in the PVN and SON, is released into the bloodstream via axonal terminals projecting to the posterior pituitary. In addition, it is suggested that oxytocin is secreted in the brain not only through dendritic or axonal release but also via paracrine mechanisms, known as volume transmission.^16^^,^^17^^,^^18^^,^^19^ Recently, our previous work also revealed how oxytocin acts through non-synaptic mechanisms in various central nervous system regions, including the lumbosacral spinal cord, to control male sexual activity.^20^^,^^21^

Previous studies investigating the connection between oxytocin and affinity have mainly focused on mother-infant relationships, particularly neural mechanisms regulating maternal behavior.^22^ Meanwhile, in social bonding beyond mother-infant relations, research has shown that oxytocin neurons are activated by social tactile stimulation,^23^ and oxytocin secretion is promoted by mutual gazing between humans and dogs.^24^ However, the underlying mechanisms of social bonding regulated by oxytocin remain elusive. In this study, we investigated the brain region mediating the mechanisms by which pleasant touch sensations, induced by gentle and repeated touch from human hands, regulate affinity from rats to human hands, focusing on the oxytocin receptor (OTR). We hypothesized that oxytocin signaling in the brain may be involved in modulating responses to pleasant touch, based on several lines of evidence: (1) OTRs are expressed in brain regions associated with social reward processing,^25^ (2) oxytocin administration enhances the rewarding properties of social stimuli,^26^ and (3) pleasant touch has been shown to activate oxytocin-synthesizing neurons in hypothalamic nuclei.^27^ To test this hypothesis, we used rats with tickling-induced high affinity for human hands and examined the role of oxytocin signaling in the ventromedial hypothalamus (VMH).

## Results

### Establishment of rats with tickling-induced high affinity for human hands through pleasant touch sensation

To investigate the mechanism underlying affinity from rats to human hands increased by tickling, we developed rats with tickling-induced high affinity for human hands. While rats typically display avoidance behaviors toward human hands, we successfully established rats with tickling-induced high affinity for human hands through repeated exposure to tickling that resembles the type of bodily contact rats obtain during rough-and-tumble play, a common social behavior among juvenile-adolescent rat peers. Tickling was administered to adolescent rats (35–45 days old) to promote affinity for human hands.

To assess the rewarding nature of the tickling procedure, we quantified 50 kHz USV during training. On day 1, rats exhibited minimal standing behavior, showed avoidance of human hands, and rarely emitted 50 kHz USV (Video S1). By day 5, rats began emitting 50 kHz USV, with emissions progressively increasing through day 10 (Figure S3). Standing behaviors indicating desire for tickling also increased in frequency (Video S2).

**Figure 1:**
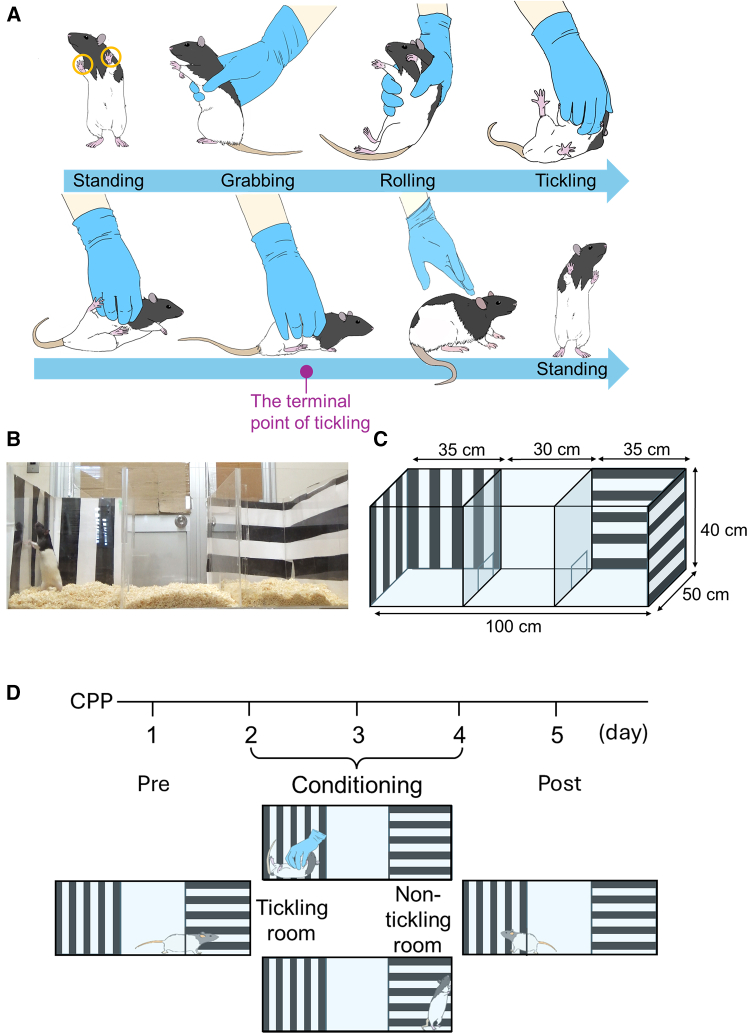
Tickling training and a CPP test (A) To assess the rat’s desire for tickling, we defined “standing” behavior as rats positioning themselves upright with both front paws placed against the wall. Tickling was administered by gently grabbing the rats from behind, carefully rolling them onto their backs, and then tickling their bellies. Rats were conditioned to receive tickling only upon exhibiting this standing behavior. (B and C) Photograph (left) and schematic diagram (right) of the CPP apparatus. (D) Experimental paradigm. The CPP test was conducted over five consecutive days. On day 1, rats underwent a pre-test to establish baseline chamber preferences. During the next 3 days, conditioning involved 5-min tickling sessions in the designated tickling room, alternating with 5-min periods in the non-tickling room. The experiment concluded with a post-test on the fifth day. See also Videos S1, S2, and S5.

Video S1. Tickling training on the first day, related to Figure 1

Video S2. Tickling training on the 10th day, related to Figure 1

**Figure 2:**
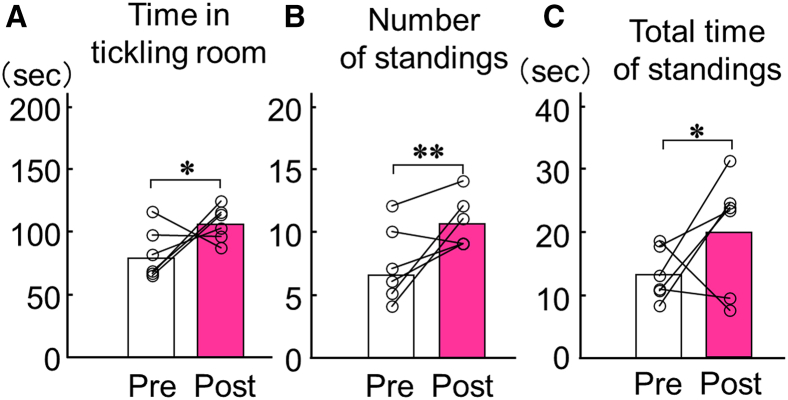
Rats developed an increased affinity for human hands through pleasurable tickling (A) CPP testing was conducted to verify whether rat’s affinity for human hands had been increased. Analysis of duration spent in each chamber revealed that rats spent significantly longer periods in the tickling room during the post-test compared with the pre-test. (B and C) Analysis of standing behaviors showed significant increases in both frequency and time spent in the tickling room during the post-test compared with pre-test. See also Figures S1–S3, Table S1, and Video S1. ^∗^*p* < 0.05, ^∗∗^*p* < 0.01, ^∗∗∗^*p* < 0.001.

Following 10 days of tickling training, we conducted conditioned place preference (CPP) tests to verify affinity from rats to human hands. Analysis of time spent in each chamber revealed that rats spent significantly longer periods in the tickling room during the post-test compared with pre-test (*p* = 0.03; Figure 2A). Analysis of standing behaviors showed significant increases in both frequency and duration in tickling room during post-test compared with pre-test (frequency: *p* = 0.005; duration: *p* = 0.047; Figures 2B and 2C). By contrast, the analysis revealed no significant difference in either time spent or standing behavior in the non-tickling room between pre-test and post-test conditions (Figure S2). Moreover, we also conducted a hand-preference test to confirm whether the increased time spent in the tickling room was induced by tickling specifically. The rats that received 10 days of tickling training (tickling group) and those that did not (control group) were tested for their preference for a room with or without a human hand (with hand room and without hand room, respectively) using the same CPP apparatus (Figures S3A and S3B). Consequently, rats showed no preference for either room irrespective of the presence or absence of tickling training for 10 days. However, the tickling group significantly increased both duration spent in with hand room and frequency of standing behavior compared with the control group (duration: *p* = 0.03; frequency: *p* = 0.04; Figures S3C and S3D).

### Enhanced OTR expression and neural activation in VMHvl of rats with tickling-induced high affinity for human hands

**Figure 3:**
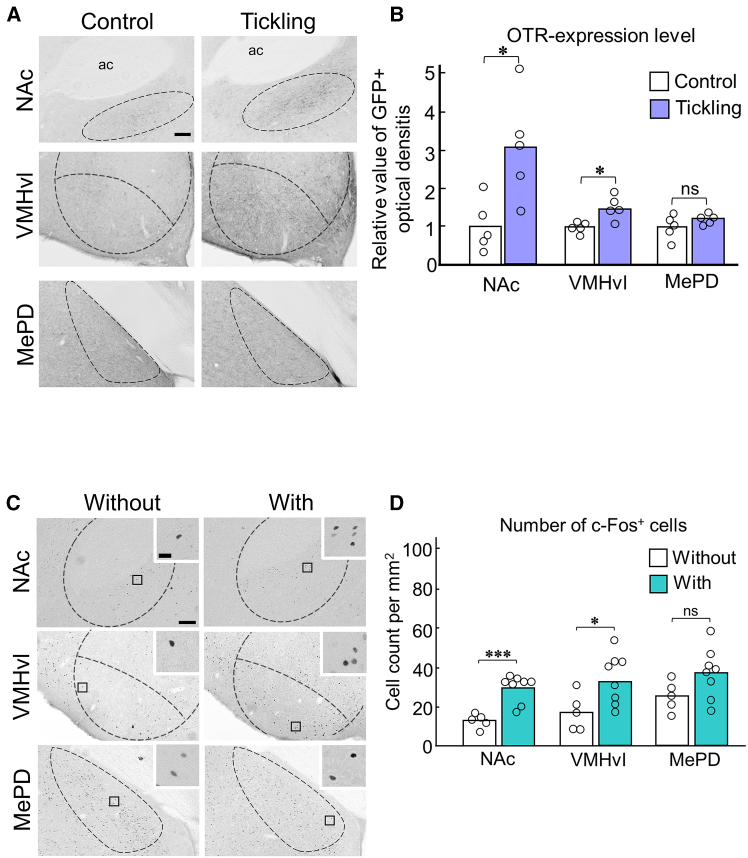
Repeated pleasant touch tickling increased the expression of OTR and c-Fos in the brain (A) Representative images of GFP immunostaining showing OTR expression levels. Scale bar, 100 μm. (B) Immunohistochemical analysis revealed significantly increased OTR expression in both the NAc and VMHvl following tickling but not in the MePD. (C) Representative images of c-Fos immunostaining. Scale bar, 100 μm; boxed areas are shown at the higher magnification, scale bar, 20 μm. (D) Tickling significantly increased c-Fos-positive cells in the NAc and VMHvl, while the MePD showed no significant changes. See also Figures S4–S6. ^∗^*p* < 0.05, ^∗∗^*p* < 0.01, ^∗∗∗^*p* < 0.001.

To identify neural substrates involved in tickling-induced high affinity from rats to human hands, we examined OTR expression levels in regions potentially influenced by repeated tickling using two experimental groups: a control group of OTR-yellow fluorescent protein (YFP)^20^ rats maintained without tickling for 10 days and a tickling group of rats receiving tickling training for 10 days (Figure S4). Immunohistochemical analysis revealed that tickling significantly upregulated OTR expression in both the nucleus accumbens (NAc) and ventrolateral part of the VMH (VMHvl) (NAc: *p* = 0.017; VMHvl: *p* = 0.013; Figures 3A and 3B). While OTR expression was also detected in the posterodorsal medial amygdala (MePD), tickling did not significantly affect expression levels in this region (*p* = 0.151; Figures 3A and 3B). In addition, we observed no significant differences in the expression level of OTR across other brain regions examined in this study (Figure S5).

**Figure 4:**
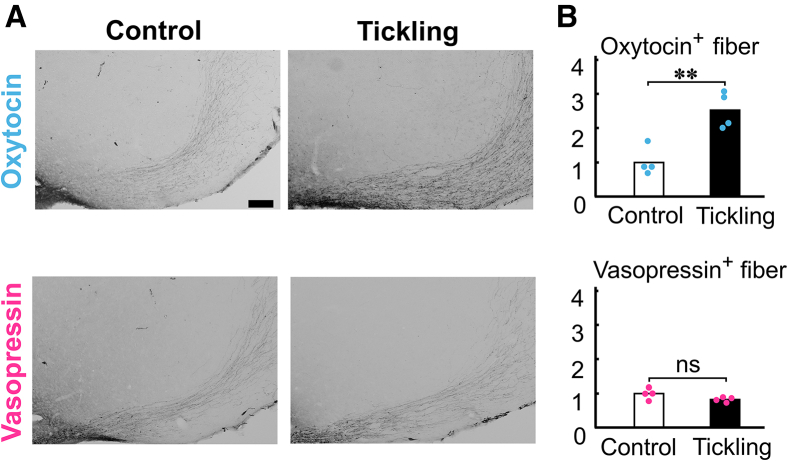
Increase in oxytocin fibers in the VMHvl following tickling (A) Representative images of oxytocin- (upper) and vasopressin- (lower) immunoreactive fibers in the VMHvl. Scale bar, 100 μm. (B) Immunohistochemical analysis revealed that the intensities of oxytocin fibers, but not vasopressin fibers, significantly increased following repeated tickling. See also Table S1.

To investigate neural activation using c-Fos immunoreactivity induced by tickling in the NAc, VMHvl, and MePD, we divided rats that underwent tickling training procedures from 5 weeks of age into two conditions: the without-tickling condition, in which rats were not subjected to tickling, and the with-tickling condition, in which rats were tickled immediately before being sacrificed (Figure S6). Tickled rats showed significantly increased c-Fos-positive cells in both the NAc and VMHvl but not in the MePD (NAc: *p* < 0.001; VMHvl: *p* = 0.022; MePD: *p* = 0.101; Figures 3C and 3D). Moreover, we compared the densities of oxytocin- and vasopressin-immunoreactive fibers in the NAc and VMHvl between tickled and non-tickled rats. Consequently, oxytocin-immunoreactive fibers were significantly increased in the VMHvl, whereas vasopressin-immunoreactive fibers remained unchanged (oxytocin: *p* = 0.004; vasopressin: *p* = 0.13; Figure 4). In the NAc, neither oxytocin- nor vasopressin-immunoreactive fibers were detected.

### Chemogenetic inhibition of VMHvl OTR neurons disrupts affinity-like behaviors

**Figure 5:**
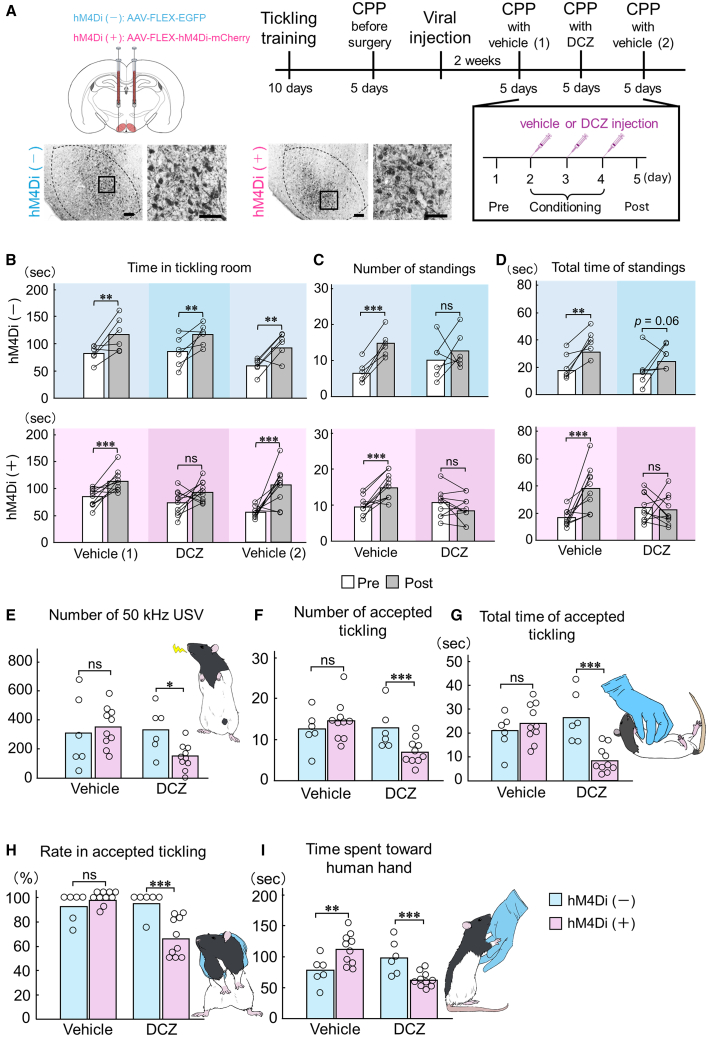
Chemogenetic inhibition of OTR neurons in the VMHvl prevented affinity-like behavior from rats to human hands that promoted pleasant touch sensation (A) To investigate the neural mechanisms underlying affinity from rats to human hands promoted by pleasant touch sensations, we performed a CPP test while inhibiting the neural activity of OTR neurons in the VMHvl. After tickling training, rats were divided into two groups: one group received an injection of an AAV-expressing GFP, while the other received an AAV-expressing hM4Di-mCherry, designated as the hM4Di(−) and hM4Di(+) groups, respectively. Following a recovery period, the rats underwent CPP testing with either DCZ, a selective ligand for hM4Di, or vehicle treatment. Successful viral infection was confirmed using immunohistochemistry for GFP or mCherry expression. Scale bars, 100 μm (left) and 50 μm (right). (B) Analysis of duration spent in each room pre-test (pre) vs. post-test (post) demonstrated that with vehicle treatment, both groups spent significantly more time in the tickling room during post compared with pre. With DCZ treatment, hM4Di(−) rats showed preference for tickling room during post compared with pre, while no difference was observed between pre and post in the hM4Di(+) group. (C) To evaluate the rats’ desire for tickling, we analyzed the frequency of standings. Vehicle treatment significantly increased standing frequency in tickling room during post compared with pre in both groups, while DCZ treatment had no effect in either group. (D) Standing duration in tickling room during post was significantly extended compared with during pre in both groups when treated with vehicle. However, DCZ-treated hM4Di(+) rats showed no increase in standing duration in tickling room during post, while hM4Di(−) rats did. (E) 50 kHz USVs did not differ between groups treated with vehicle. However, DCZ treatment significantly inhibited 50 kHz USV in the hM4Di(+) group only. (F and G) Analysis of standing frequency and duration demonstrated that DCZ treatment inhibited standing behavior in the hM4Di(+) group. (H) In DCZ-treated hM4Di(+) rats, the frequency of rejection-like behavior toward tickling increased despite displaying standing behavior, leading to a significant decrease in tickling success rate. (I) Rats frequently initiated contact with the human hand through behaviors such as sniffing, licking, huddling, and rearing to climb onto the hand. These behaviors were measured as time spent oriented toward human hand, which significantly decreased only in the DCZ-treated hM4Di(+) group. See also Figure S7, Table S1, and Videos S3 and S4. ^∗^*p* < 0.05, ^∗∗^*p* < 0.01, ^∗∗∗^*p* < 0.001.

Based on the coincident elevation of OTR expression and neural activation in the VMHvl, we examined the role of VMHvl OTR neurons in affinity-like behaviors from rats to human hands using chemogenetic inhibition during CPP testing. After initial tickling training, OTR-Cre rats^28^ were confirmed to have increased affinity for human hands using CPP. Then, the rats that showed high affinity for human hands were divided into two groups: hM4Di(−) expressing green fluorescent protein (GFP) only and hM4Di(+) expressing hM4Di-mCherry. 2 weeks after adeno-associated virus (AAV) vector injection, CPP testing was conducted with the administration of either vehicle or deschloroclozapine (DCZ), a selective hM4Di ligand (Figure 5A).

Vehicle-treated rats in both groups showed significantly increased time spent in the tickling room during post-test (hM4Di(−): *p* = 0.001; hM4Di(+): *p* < 0.001). After DCZ administration, hM4Di(−) rats maintained their preference for tickling room (*p* = 0.01), while hM4Di(+) rats showed no significant preference (*p* = 0.086; Figure 5B).

Analysis of standing behavior revealed that both groups showed significantly increased frequency and duration of standings in the tickling room during post-test with vehicle treatment (frequency: hM4Di(−): *p* < 0.001; hM4Di(+): *p* < 0.001; duration: hM4Di(−): *p* = 0.001; hM4Di(+): *p* < 0.001; Figures 5C and 5D). However, hM4Di(+) rats treated with DCZ did not show a significant increase in the duration of standings in the tickling room during post-test compared with pre-test (*p* = 0.738; Figures 5C and 5D). To assess whether rats desired to contact the familiar human hand, we examined behaviors during conditioning. This revealed that the 50 kHz USV indicating pleasure did not differ between vehicle-treated hM4Di(−) and hM4Di(+) rats and that 50 kHz USV decreased only in the hM4Di(+) rats following DCZ treatment (*p* = 0.001; Figure 5E). Moreover, the frequency and duration of received tickling were significantly reduced only in the hM4Di(+) group following DCZ treatment (frequency: *p* < 0.001; duration: *p* < 0.001; Figures 5F and 5G). While the hM4Di(+) rats displayed standing behavior associated with tickling, they also occasionally showed rejection-like behavior toward tickling. In this case, it was impossible to roll and tickled rats (Figure S7; Video S3). Because the frequency of rejection-like behavior toward tickling increased despite the rats displaying standing behavior, the rate of accepted tickling (number of accepted ticklings divided by total standings) significantly decreased (*p* < 0.001; Figure 5H). Furthermore, all the experimental rats frequently exhibited behaviors indicating affinity for human hands, such as licking, huddling, and clinging to the experimenter’s hand (Video S4). This behavior, defined as “time spent toward human hand,” was significantly reduced only in the DCZ-treated hM4Di(+) rats (*p* < 0.001; Figure 5I).

**Figure 6:**
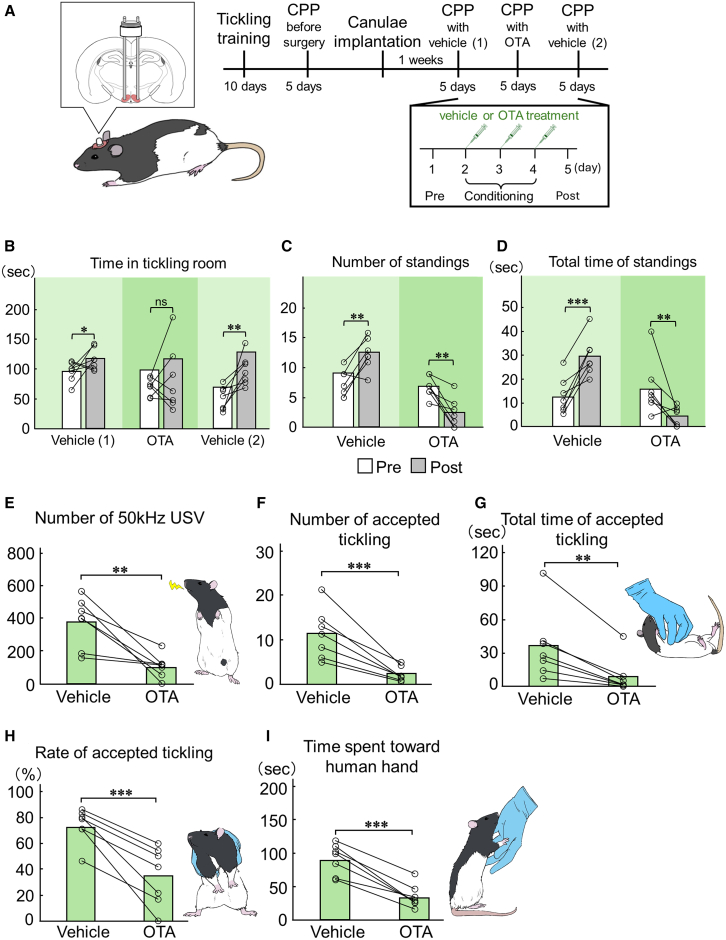
OTR antagonism in the VMHvl prevented affinity-like behaviors from rats to human hands promoted by pleasant touch sensation (A) To directly assess oxytocin’s role in VMHvl-mediated affinity for human hands, we conducted CPP testing with local administration of an OTA to the VMHvl. Rats exhibiting a high affinity for human hands were implanted with guide cannulae in the VMHvl and underwent CPP testing with vehicle or OTA treatment. (B) Analysis of duration spent in tickling room demonstrated that vehicle treatment significantly increased time spent in tickling room during post compared with pre, while OTA treatment eliminated preference for tickling room. (C and D) Standing behavior analysis showed that vehicle treatment increased both frequency and duration of standings during post, while OTA treatment significantly reduced both measures. | (E) 50 kHz USVs significantly decreased as a result of OTA treatment compared with vehicle treatment. (F–H) Standing frequency, duration, and success rate significantly decreased as a result of OTA treatment compared with vehicle. (I) Time spent oriented toward the human hand significantly decreased as a result of OTA treatment. See also Figure S7, Table S1, and Video S3. ^∗^*p* < 0.05, ^∗∗^*p* < 0.01, ^∗∗∗^*p* < 0.001.

Video S3. Tickling-rejection-like behavior, related to Figures 5 and 6

Video S4. Time toward human hands, related to Figure 5

### OTR antagonism in the VMHvl reduces affinity-like behaviors

To directly assess oxytocin’s role in VMHvl-mediated affinity from rats to human hands, we conducted CPP testing with local administration of an OTR antagonist (OTA) to the VMHvl. After initial tickling training, rats were confirmed to have an increased affinity for human hands using CPP. Subsequently, rats exhibiting a high affinity for human hands had implanted guide cannulae into the VMHvl. Following a 1-week recovery period, CPP was conducted with the administration of either vehicle or OTA (Figure 6A). Vehicle-treated rats showed significant preference for the tickling room (*p* = 0.039; Figure 6B), while OTA administration eliminated this preference (*p* = 0.264; Figure 6B).

Standing behavior analysis showed that vehicle treatment increased both the frequency and duration of standing during the post-test period (frequency: *p* = 0.009; duration: *p* < 0.001; Figures 6C and 6D). OTA treatment showed significant reductions in the frequency and duration of both measures (frequency: *p* = 0.003; duration: *p* = 0.003; Figures 6C and 6D). We also examined behaviors during conditioning. OTA treatment significantly reduced 50 kHz USV frequency compared with vehicle treatment (*p* = 0.004; Figure 6E). Moreover, OTA-treated rats showed a significant reduction in frequency, duration, and rate of accepted tickling (frequency: *p* < 0.001; duration: *p* = 0.002; rate: *p* < 0.001; Figures 6F–6H). The time spent toward the human hand was also significantly reduced by OTA treatment (*p* < 0.001, Figure 6I).

### Identification of oxytocin neural circuits in the VMH

**Figure 7:**
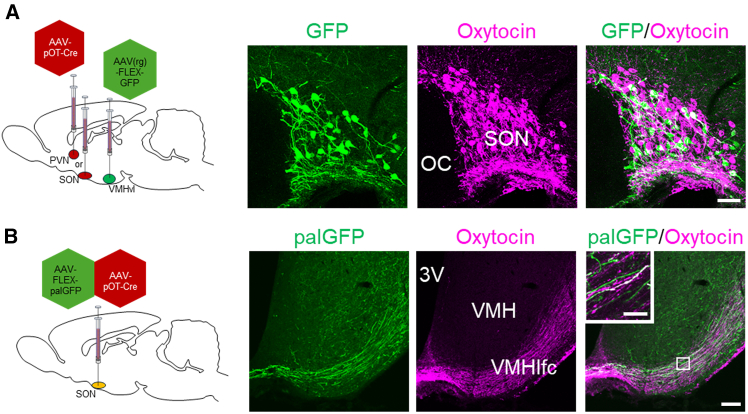
Identification of oxytocin neurons projecting to the VMH (A) AAV-expressing Cre recombinase specifically in oxytocin neurons was injected into the PVN or the SON, while AAV-expressing Cre-dependent GFP was injected into the VMHvl for retrograde labeling. This retrograde marking demonstrated that oxytocin neurons projecting to the VMH region were labeled by GFP-immunoreactive cells in the SON. Scale bar, 100 μm. (B) To visualize axonal projections from oxytocin neurons in the SON and PVN that are present to the VMH, we performed anterograde labeling using AAV-expressing palGFP. Within the SON, a subset of magnocellular oxytocin neurons showed palGFP expressions, and their axonal projections were detected in the VMHlfc. Scale bar, 100 μm; the boxed area is shown at the higher magnification. Scale bar, 20 μm.

To elucidate the neural circuit mediating oxytocin release in the VMH, we conducted retrograde and anterograde tracing studies focusing on the PVN and SON, primary sources of oxytocin. Retrograde tracing revealed GFP-immunoreactive oxytocin neurons in the SON after VMH (Figure 7A). However, identical injections revealed no GFP-immunoreactive neurons in the PVN. Anterograde tracing showed palGFP-positive oxytocin neurons in the SON with positive processes within the VMH lateral fiber complex (VMHlfc) (Figure 7B), suggesting direct projections from magnocellular SON oxytocin neurons to the VMH.

These findings collectively demonstrate that oxytocin signaling in the VMHvl, potentially mediated by projections from SON oxytocin neurons, plays a crucial role in the establishment and maintenance of affinity-like behaviors induced by pleasant touch sensation with human hands.

## Discussion

In this study, we demonstrate that tickling-induced affinity from rats to human hands is mediated by OTR fibers in the VMHvl. Using chemogenetic inhibition of OTR-expressing neurons and pharmacological blockade of OTRs in the VMHvl, we show that release of oxytocin from these oxytocin fibers is essential for both CPP for tickling-associated environments and approach behaviors toward human hands. Furthermore, our tracing studies suggest that magnocellular oxytocin neurons in the SON project to the VMHvl, potentially utilizing *en passant* volume transmission to regulate social bonding between rats and humans.

The rats with tickling-induced high affinity for human hands were subjected to 10 days of tickling during their juvenile-adolescent period and exhibited increased emission of 50 kHz USV. Our tickling protocol resembles the type of bodily contact rats obtain during rough-and-tumble play, a natural behavior observed among juvenile-adolescent rats with their peers.^29^^,^^30^ Since rats emit 50 kHz USVs during social play, this suggests that rough-and-tumble play provides pleasant touch sensation.^4^^,^^31^ Multiple studies have demonstrated that rats emit 50 kHz USVs and display positive emotional responses when tickled.^5^^,^^32^^,^^33^ In our study, rats appeared to experience tickling by human hands as a pleasant touch sensation after repeated exposure and developed a high affinity for human hands. This was confirmed by CPP testing, where rats showed a clear preference for the tickling-associated room, indicating the rewarding nature of the interaction. Furthermore, when rats were trained to receive tickling contingent on displaying standing behavior, they actively sought additional tickling interactions. Consistent with previous research showing that pleasant touch sensations promote contact with human hands in rats,^32^ we observed increased approach behavior toward human hands as a result of tickling. Given that pleasant touch sensation is fundamental to affinity-like behaviors,^1^ our findings suggest that human touch-induced play behavior incorporating pleasant touch sensation facilitated an increase in the rat’s affinity for human hands.

The tickling protocol served dual purposes: providing pleasant touch sensation and acting as a social stimulus through contact with another individual. Oxytocin is well-established as a mediator of various social behaviors,^34^ with affinity-like behaviors—our primary focus—being a crucial example.^35^^,^^36^ Our results reveal that the brain’s oxytocin system plays an essential role in increasing rat’s affinity for human hands. Rats that developed a high affinity for human hands following repeated tickling exhibited significantly higher OTR expression in the VMHvl compared with control rats that showed a low affinity for human hands. Further investigation using either Designer Receptors Exclusively Activated by Designer Drugs (DREADDs)-mediated inhibition of OTR-expressing neurons or OTA administration in the VMHvl demonstrated that suppressing OTR activity inhibited both CPP for the tickling-associated room and affinity-like behaviors from rats to human hands, including 50 kHz USVs and approach behavior (Figures 5 and 6). Our findings demonstrate that DREADD-mediated inhibition of OTR-expressing neurons in the VMHvl and direct OTR antagonism produced similar behavioral outcomes, suggesting that both neuronal activation and OTR signaling in this region are crucial for the development of affinity-like behavior. Previous studies have shown that oxytocin neurons in the SON are activated during pleasant touch from physical contact,^37^^,^^38^ and pleasant touch sensation is enhanced by oxytocin administration.^39^ Our findings suggest that disruption of the OTR-VMHvl system reduces the pleasurable aspects of human touch-induced play behavior.

Mouse CPP studies have demonstrated that preference for social interaction is eliminated by OTA administration,^40^ consistent with oxytocin’s established role in regulating social motivation.^41^ Our findings suggest that suppressed affinity-like behavior from rats to human hands resulting from inhibited oxytocin activity in the VMHvl reflects reduced pleasure associated with social touch from human hands.

The VMHvl has been implicated in various social behaviors, including aggression, mating, and social recognition. Previous studies have shown that changes in OTR expression in this region modulate these behaviors, particularly in the context of sexual receptivity and maternal behaviors. Our findings extend this understanding by demonstrating that OTR signaling in the VMHvl also mediates tickling-induced affinity for human hands, suggesting a broader role for this system in cross-species social bonding. The involvement of OTR in the VMHvl in both CPP and approach behaviors indicates its role in processing rewarding aspects of social touch, consistent with previous findings linking OTR signaling to social reward processing.

Our investigation using oxytocin neurophysin I (NPI) immunostaining (with PS60 antibody) to label oxytocin fibers revealed that in addition to the dense axonal projections in the VMHlfc, we observed sparse SON→VMH palGFP single-positive fibers within the VMH (Figure 7B). This observation suggests that palGFP may visualize processes of oxytocin neurons containing few or no oxytocin-containing neurosecretory vesicles, whereas the PS60 antibody specifically identifies oxytocin fibers containing neurosecretory vesicles. The higher prevalence of palGFP single-positive fibers within the VMHvl suggests that a subset of magnocellular SON neurons may project to or terminate in the VMHvl. These axonal fibers might contain fewer oxytocin-containing neurosecretory vesicles or release oxytocin more frequently, potentially explaining their weak or absent PS60-immunoreactivity within the VMHvl. The increased oxytocin-ir fiber density in the VMHvl following repeated tickling could have multiple functional implications. This increase might reflect enhanced capacity for oxytocin release, potentially supporting stronger social bonding. Alternatively, it could indicate the accumulation of oxytocin within fibers due to reduced release, which would have different behavioral consequences. While vasopressin fibers remain present and unchanged in density, our pharmacological evidence supports a specific role for OTR signaling in tickling-induced affinity. However, as noted in the field, the relationship between fiber density and neuropeptide release remains an important unresolved question.

Through AAV vector tracing techniques, we demonstrated that magnocellular oxytocin neuronal axons from the SON traverse the VMHlfc *en route* to the posterior pituitary. Within the VMH, OTR-expressing neurons are specifically concentrated in its ventrolateral part (VMHvl). Oxytocin released in the VMHlfc likely diffuses and readily accesses the VMHvl, reflecting the rational molecular and anatomical organization of the oxytocin-OTR system in the VMH, which, however, requires further study. While we did not observe GFP-positive oxytocin neurons in the PVN, which is an important region during social interaction, following our retrograde tracing in this study, we cannot completely exclude their potential contribution to controlling the oxytocin-OTR system in the VMH, as viral tracing techniques may have inherent limitations in detection sensitivity. While retrograde AAVs are primarily taken up at synaptic terminals, evidence suggests they can also be internalized along axons,^42^^,^^43^ which may explain our observation of labeled oxytocinergic fibers in the median eminence. Nevertheless, it is important to note that our hypothesis focused on magnocellular neurons. The morphological signature of *en passant* volume transmission of oxytocin from SON magnocellular axons may provide a novel mechanism of peptide spread through the brain tissue and regulate various behaviors.

Notably, while the specific release site of oxytocin (whether from dendrites, cell bodies, axons, or synapses) may be less critical, the regulation of *en passant* release mechanisms remains to be fully elucidated. Action potentials are not necessarily required for dendritic oxytocin secretion,^16^ raising important questions about the temporal and spatial control of *en passant* oxytocin release. Given appropriate molecular machinery for exocytosis, localized non-synaptic release independent of action potentials may be possible. CD38, an established oxytocin-releasing factor,^44^ represents a potential key molecule in this process. Additionally, CD38 regulates insulin exocytosis in pancreatic islets through ATP-dependent mechanisms.^45^ The local availability of mitochondrial ATP, CD38, and various soluble *N*-ethylmaleimide sensitive factor attachment protein receptor (SNARE) complexes may be necessary for *en passant* non-synaptic release of oxytocin from axonal varicosities. Previous research has demonstrated that the vesicle-SNARE protein synaptotagmin IV is specifically expressed in oxytocin-containing neurosecretory vesicles but not in vasopressin-containing vesicles, suggesting its crucial role in oxytocin exocytosis.^46^ Consequently, synaptotagmin IV may specifically participate in *en passant* oxytocin release. Further investigations are necessary to establish definitive conclusions regarding these mechanisms.

### Conclusions

Our study demonstrates for the first time that oxytocin signaling in the VMHvl is critical for human touch-induced play behavior in rats. Using a combination of behavioral, pharmacological, and chemogenetic approaches, we showed that OTR activation in VMHvl neurons is essential for rats to develop positive responses to tickling stimulation, as evidenced by 50 kHz USVs, approach behaviors, and CPP. We found that either chemogenetic inhibition of OTR neurons or local OTR antagonism in the VMHvl not only prevented the expression of tickling-induced play behaviors but also diminished the rats’ engagement in playful interactions with human hands. Additionally, we identified direct projections from the SON to the VMH that likely modulate this playful social behavior. Furthermore, our morphological analysis suggests a novel mechanism of oxytocin action: magnocellular oxytocin neurons projecting to the posterior pituitary may release oxytocin within the hypothalamus through non-synaptic *en passant* volume transmission. This hitherto unrecognized mode of oxytocin release potentially controlling human touch-induced play behavior provides new insights into how pleasant touch promotes social affinity through the oxytocin system. Together, this work advances our understanding of how oxytocin shapes social behavior and may inform the development of therapeutic strategies to promote positive social interactions.

## Resource availability

### Lead contact

Requests for resources and reagents should be directed to and will be fulfilled by the lead contact, Hirotaka Sakamoto (hsakamo@okayama-u.ac.jp).

### Materials availability

Research materials generated in this study are available from the corresponding author upon request.

### Data and code availability

• No standardized datasets were generated during this study.
• The published article includes all datasets analyzed during this study.
• No original code was used in this study.

## Acknowledgments

This work was supported by Grants-in-Aid for Scientific Research from the Japan Society for the Promotion of Science (JSPS) KAKENHI, Japan (to H.S.; 22H02656 and 22K19332); by the Takeda Science Foundation, Japan (to H.S., Bioscience Research Grants); by the Naito Foundation, Japan (to H.S.; Naito Memorial Grant for Natural Science Researchers); by the Ryobi Teien Memory Foundation, Japan (to H.S.; Research Grants); by the Wesco Scientific Promotion Foundation, Japan (to H.S., International Travel Grants; Research Grants); by the TOYO SUISAN Foundation, Japan (to H.S.; Research Grants); and by the Japan Foundation for Applied Enzymology, Japan (to H.S.; Research Grants). H.H. is supported by the JST SPRING, Japan grant number JPMJSP2126. The authors would like to thank Professor John F. Morris (University of Oxford, UK) for critically reading the manuscript. This work was supported by the Synergy European Research Council (ERC) grant “OxytocINspace” 101071777, SFB Consortium 1158-3, and German-Israeli Project cooperation (DIP) GR3619-1 to V.G. The illustration was created by Yuto Inai, Professional Japanese Manga Artist.

## Author contributions

H.H. performed behavior analyses under the supervision of Y.S. H.H., S.T., and S.M. performed histological experiments and surgery. A.I. and T.O. designed and produced the AAV vectors. D.H. and V.G. provided advice, gene-modified animals, and equipment. H.H. and H.S. wrote the paper. The whole study was supervised by H.S. All authors discussed the results and commented on the manuscript.

## Declaration of interests

The authors declare no competing interests.

## STAR★Methods

### Key resources table

**Table:** 

REAGENT or RESOURCE	SOURCE	IDENTIFIER
**Antibodies**		

Chicken Anti-GFP	Rockland	600-901-215; RRID: AB_1537402
Rabbit Anti-c-Fos	Abcam	ab190289; RRID: AB_2737414
Mouse Anti-NPI (PS60)	ATCC	CRL-1800; RRID: AB_2722605
Mouse Anti-NPII (PS40)	ATCC	CRL-1799; RRID: AB_2313960
Chicken Anti-mCherry	Abcam	ab205402; RRID: AB_2722769

**Chemicals, peptides, and recombinant proteins**		

d (CH2)51, Tyr(Me)2, Thr4, Orn8, Tyr-NH29)-vasotocin trifluoroacetate	Bachem	H-9405
deschloroclozapine	Selleck Chemicals	Cat# E1265; CAS# 1977-07-7

**Experimental models: Organisms/strains**		

Wistar	Charles River	N/A
Long-Evans	Japan SLC, Inc.	N/A
W-Tg*(*pOxtr-HB-EGF-2A-ChR2-YFP*)1Hs*	Oti et al.^20^	https://doi.org/10.1016/j.cub.2020.09.089
LE;SD-*Oxtr*^*tm1(IRES-Cre)*^	Iwasaki et al.^28^	https://doi.org/10.1038/s41467-023-36641-7

**Recombinant DNA**		

AAV(DJ)-hSyn-FLEX-hM4Di-mCherry	This paper	N/A
AAV(Sr9)-hSyn-FLEX-EGFP-WPRE	This paper	N/A
AAV(DJ)-pOT-Cre-WPRE	Sakamoto and Inutsuka^47^	https://doi.org/10.1267/ahc.24-00001
AAV(rg)-FLEX-GFP	This paper	N/A
AAV(Sr9)-CAG-FLEX-palGFP-WPRE	Inutsuka et al.^48^	https://doi.org/10.1038/s42003-022-03944-2

**Software and algorithms**		

ImageJ Software	NIH	RRID:SCR_003070
BellCurve for Excel	BellCurve	N/A
cellSensSoftware	Olympus	RRID:SCR_016238

**Other**		

Double cannulae guide canulae	RWD Life Science	Cat#62030
Double cannulae dummy canulae	RWD Life Science	Cat#62130
Double cannulae Injector	RWD Life Science	Cat#62230
Double cannulae cap	RWD Life Science	Cat#62523

### Experimental model and subject details

#### Rats

Male Long-Evans rats (*N* = 46, Japan SLC, Inc., Shizuoka, Japan) and Wistar rats (*N* = 10, Charles River, Yokohama, Japan) were used as wild-type subjects. Additionally, male OTR promoter-human heparin-binding epidermal growth factor-like growth factor human diphtheria toxin receptor (hDTR)-channelrhodopsin-2 (ChR2)-yellow fluorescent protein (YFP) BAC transgenic rats (*N* = 10, referred to as OTR-YFP rats; Wistar strain)^20^ and OTR-IRES-Cre knock-in rats (*N* = 16, referred to as OTR-Cre rats),^28^ backcrossed for at least five generations onto the Long-Evans background, were bred in the Okayama University Animal Facilities, Japan. All rats were housed in pairs under a 12-hour light/dark cycle with ad libitum access to water and standard rodent chow. All experimental procedures were conducted in accordance with protocols approved by the Animal Experiment Committees of Okayama University, Japan.

### Method details

#### Behavioral Experiments

##### Tickling training

Beginning at 5 weeks of age, rats underwent daily tickling training in a cage equivalent in size to their home cage while measuring 50 kHz ultrasonic vocalizations (USV). Initially, the experimenter’s hand which wear blue surgical glove, was placed motionless in the training cage to facilitate habituation. Subsequently, tickling was introduced, consisting of grabbing the rats from behind, rolling them onto their backs, and tickling their bellies (Figure 1A; Video S5). To assess tickling desire, we defined "standing" behavior as rats standing upright with both front paws placed on the cage wall. Rats were conditioned to receive tickling only upon exhibiting this standing behavior. Tickling was terminated if rats twisted their body to right themselves or resisted rolling by bracing their hind legs. Training sessions lasted 10 minutes daily for 10 consecutive days.

Video S5. Tickling training methods, related to Figure 1

##### Conditioned place preference test

The conditioned place preference test (CPP) was conducted in an acrylic apparatus (100 × 50 × 40 cm) divided into three rooms, with the left and right rooms distinguished by vertical or horizontal stripes (Figure 1B). Square holes (8 × 8 cm) in the partition walls allowed free movement between rooms. This experimental design was refined through preliminary analysis, indicating that a two-day conditioning protocol was inadequate for eliciting the desired tickling response. The CPP protocol consisted of five consecutive days: pre-test (Pre), three conditioning days, and post-test (Post) (Figure 1D).

During the pre-test, rats freely explored the apparatus for 5 minutes while time spent, number of standings, and standing duration in each striped room were recorded. Based on pre-test results, the less-preferred room was designated as the tickling room and the more-preferred room as the non-tickling room. During conditioning, partition holes were blocked to confine rats to specific rooms. Rats in the tickling room received 5 minutes of tickling using the training protocol, while rats in the non-tickling room remained unstimulated for 5 minutes regardless of standing behavior. Daily conditioning sessions totaled 10 minutes (5 minutes per room) for three consecutive days. In the post-test, rats freely explored the apparatus for 5 minutes with partition holes open, and time spent in tickling and non-tickling room was measured and compared to pre-test results.

All behavioral tests were video-recorded and analyzed using Event Recorder software. For pre- and post-tests, we measured time spent, number of standings, and standing duration in each room. During conditioning, we measured the number of tickling instances, total tickling time (duration from standing initiation to completion of self-righting), tickling success rate (number of tickling divided by total standings), and time spent contact with gloved human hand (total duration of rat-initiated contact including sniffing, licking, huddling, and rearing to cling).

#### Stereotaxic surgeries

##### Viral injection

All adeno-associated virus (AAV) vectors were produced using the AAV Helper-Free System (Agilent Technologies, Inc., Santa Clara, CA, USA) and purified by established methods.^48^ Stereotaxic surgery for AAV injections was performed under 2% isoflurane anesthesia. All AAVs were bilaterally injected at 1 μL per side. For chemogenetic inhibition of OTR neurons in the VMH, we injected AAV(DJ)-hSyn-FLEX-hM4Di-mCherry (experimental group) or AAV(Sr9)-hSyn-FLEX-EGFP-WPRE (control group) into the VMH (± 0.9 mm lateral, –2.7 mm posterior to bregma, +9.0 mm below skull surface).

The oxytocin mini-promoter (pOT) sequence was used to specifically label oxytocin neurons. For retrograde tracing, AAV(DJ)-pOT-Cre-WPRE^47^ was injected bilaterally into either the PVN (± 0.6 mm lateral, –1.85 mm posterior to bregma, +7.8 mm below skull surface) or the SON (± 1.8 mm lateral, –1.4 mm posterior to bregma, +8.8 mm below skull surface), while AAV(rg)-FLEX-GFP was simultaneously injected bilaterally into the VMH. For anterograde tracing, we injected a viral cocktail containing AAV(DJ)-pOT-Cre-WPRE and AAV(Sr9)-CAG-FLEX-palGFP-WPRE^48^ into either the PVN or SON. A vector coding palGFP was a kind gift from Dr. Takahiro Furuta (Osaka University, Japan).

##### Double cannula implantation

For oxytocin receptor antagonist (OTA) administration, stainless steel guide cannulae (Catalog No. Guide canulae: 62030, Dummy canulae: 62130, Injector: 62230, Cap: 62523; RWD Life Science, San Diego, USA) were implanted bilaterally into the VMH (± 0.9 mm lateral, –2.7 mm posterior to bregma, +9.0 mm below skull surface) under 2% isoflurane anesthesia. Cannulae were secured to the skull using dental acrylic cement (Matsukaze Co., Ltd., Kyoto, Japan) and anchored with surgical screws. Dummy cannulae were inserted to prevent clogging, and customized caps were attached. Rats recovered for at least one week before post-surgical CPP testing.

##### Experimental interventions

Two to three weeks after virus injection, OTR neurons in the VMH were chemogenetically inhibited. Twenty minutes before the conditioning phase on Days 2–4 of CPP, rats received intraperitoneal (i.p.) injections of either deschloroclozapine (DCZ, Selleck Chemicals, Houston, USA; 0.05 mg/0.2 mL dissolved in 5% DMSO saline) as the hM4Di ligand or vehicle control.

One week after cannula implantation, rats received bilateral microinjections of OTA (d (CH_2_)_5_^1^, Tyr(Me)^2^, Thr^4^, Orn^8^, Tyr-NH_2_^9^)-vasotocin trifluoroacetate, Bachem AG, Bubendorf, Switzerland) dissolved in 5% DMSO saline. Using double cannula microinjectors, 1 μg/1 μL of OTA or vehicle control was administered bilaterally 20 minutes before the conditioning phase on Days 2–4 of CPP.

#### Histological analyses

##### Tissue preparation

Rats were deeply anesthetized with isoflurane and perfused intracardially with heparinized 0.9% saline followed by 4% paraformaldehyde in 0.1 M phosphate buffer (PB; pH 7.4). Brains were immediately removed and post-fixed overnight in the same fixative, then transferred to 25% sucrose in 0.1 M phosphate-buffered saline (PBS) for 48 hours. Brain sections were prepared at 30-μm thickness using a cryostat (CM3050 S, Leica, Nussloch, Germany).

##### Immunohistochemistry

To visualize YFP signals indicating OTR expression levels, sections from OTR-YFP rats underwent immunohistochemistry. Sections were washed three times with PBS and incubated in 1% H_2_O_2_, 60% methanol for 20 minutes. After washing with PBS, the sections were blocked in TNGS (0.3% Triton X-100, 1% normal goat serum, and 1% bovine serum albumin in PBS) at room temperature for 30 minutes. The sections were then incubated with anti-GFP antibody (chicken polyclonal, 1:2,000; 600-901-215, Rockland, RRID: AB_1537402) in TNGS at room temperature for 1 hour, followed by overnight incubation at 4°C. Immunoreactivity was visualized using a streptavidin-biotin kit (Nichirei, Tokyo, Japan) and 3,3’-diaminobenzidine substrate solution containing 0.02% nickel chloride.

For c-Fos, oxytocin neurophysin I (NPI) and vasopressin neurophysin II (NPII) expression analysis, we performed immunohistochemistry using anti-c-Fos antibody (rabbit polyclonal, 1:10,000; ab190289, Abcam, RRID: AB_2737414), anti-NPI antibody (mouse monoclonal; PS60, 1:2,000; CRL-1800, ATCC, RRID: AB_2722605) and anti-NPII antibody (mouse monoclonal; PS41, 1:2,000; CRL-1799, ATCC, RRID: AB_2313960) following the same protocol. To confirm AAV infection in chemogenetic inhibition experiments, we conducted immunostaining for fluorescent proteins using anti-mCherry antibody (chicken polyclonal, 1:2,000; ab205402, Abcam, RRID: AB_2722769) and anti-GFP antibody (Figure 5A).

For tracing studies, two to three weeks after AAV injection, rats were processed for histological analyses. We performed double immunostaining for GFP and NPI. Following washing and blocking steps, sections were co-incubated with anti-GFP and anti-NPI antibodies (PS60) in TNGS at room temperature for 1 hour, followed by overnight incubation at 4°C. Sections were then incubated for 1 hour at room temperature with Alexa Fluor 488-linked goat anti-chicken IgY (A11034, Molecular Probes, AB_2576217) and Alexa Fluor 546-linked anti-mouse IgG (A11030, Molecular Probes, AB_2737024). All immunoreacted sections were imaged using a confocal laser scanning microscope (FV1000, Olympus, Tokyo, Japan).

##### Image analyses

GFP, NPI and NPII immunoreactive cells and fibers were analyzed using ImageJ (Version 1.45p; NIH). Individual data points were derived by averaging at least 10 photographs per area. Areas were measured and converted to relative values, with control group values set to ‘1’ for comparison with experimental groups.

### Quantification and Statistical Analysis

#### Statistics

All analyses were performed using Excel Statistics software. For CPP analysis, time spent, number and duration of standings in the 2 tests (Pre and Post) × 2 rooms (tickling room and non-tickling room were examined by two-way analysis of variance (ANOVA) with repeated measures, followed by multiple comparisons with Bonferroni tests. Effect sizes were calculated using Cohen’s d test. The behaviors during conditioning of a single CPP were averaged over the three-day period.

For chemogenetic inhibition experiments, tickling numbers, total time, success rate, time spent toward human hand, and 50 kHz USV across 3 tests [vehicle(1), DCZ, and vehicle(2)] × 2 groups [hM4Di(–) and hM4Di(+)] were analyzed using two-way ANOVA with repeated measures, followed by Bonferroni multiple comparisons. For OTA administration experiments, behaviors during conditioning across the 3 tests [vehicle(1), OTA, and vehicle(2)] were examined using one-way ANOVA with repeated measures, followed by Bonferroni multiple comparisons. Student’s t-tests were employed to analyze c-Fos expression, oxytocin fiber density, and OTR expression levels. All statistical data sets were summarized in the table (Table S1).

## Supplemental information

Document S1. Figures S1–S7 and Table S1

Document S2. Article plus supplemental information
