# Supplementary material for: Oxytocin facilitates human touch-induced play behavior in rats
Source: Curr Biol. 2025 Jun 23;35(12):2916–2926.e3. doi: 10.1016/j.cub.2025.05.034 (PMC12201962; doi:10.1016/j.cub.2025.05.034)
Supplement: Document S1. Figures S1–S7 and Table S1 [file mmc1.pdf]

Current Biology, Volume *35*

Supplemental Information

Oxytocin facilitates human touch-induced play behavior in rats

Himeka Hayashi, Sayaka Tateishi, Ayumu Inutsuka, Sho Maejima, Daisuke Hagiwara, Yasuo Sakuma, Tatsushi Onaka, Valery Grinevich, and Hirotaka Sakamoto

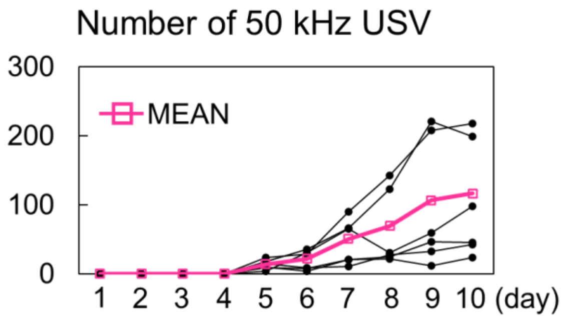

Figure S1. Progressive Increase in 50 kHz Ultrasonic Vocalizations (USV) During Tickling Training, Related to Figure 2.

Rats showed minimal 50 kHz USV emission on the first day of tickling training. Such emissions began to increase by day 5 and continued to increase progressively through day 10 of training.

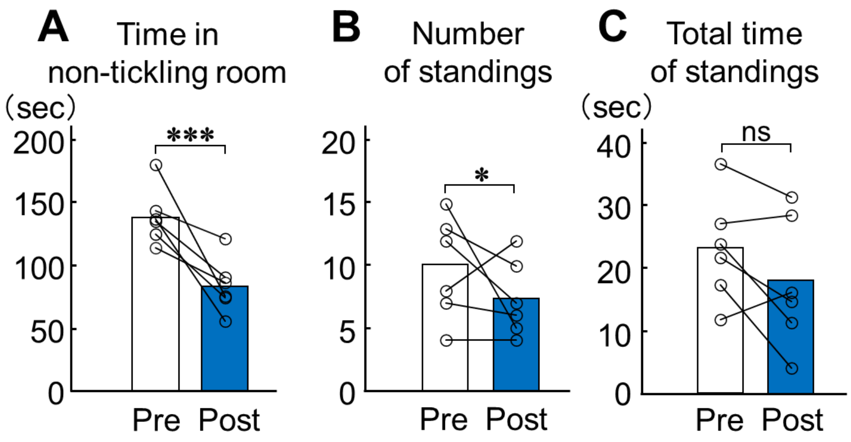

Figure S2. Behavioral Changes in the Non-Tickling Room Following Increased Affinity for Human Hands through Pleasurable Tickling, Related to Figure 2.

(A) Time spent in the non-tickling room was significantly decreased in the post-test (Post) compared to the pre-test (Pre). (B, C) In contrast to the behavior in the tickling room, rats showed fewer standings and unchanged standing duration during the post-test compared to the pre-test.

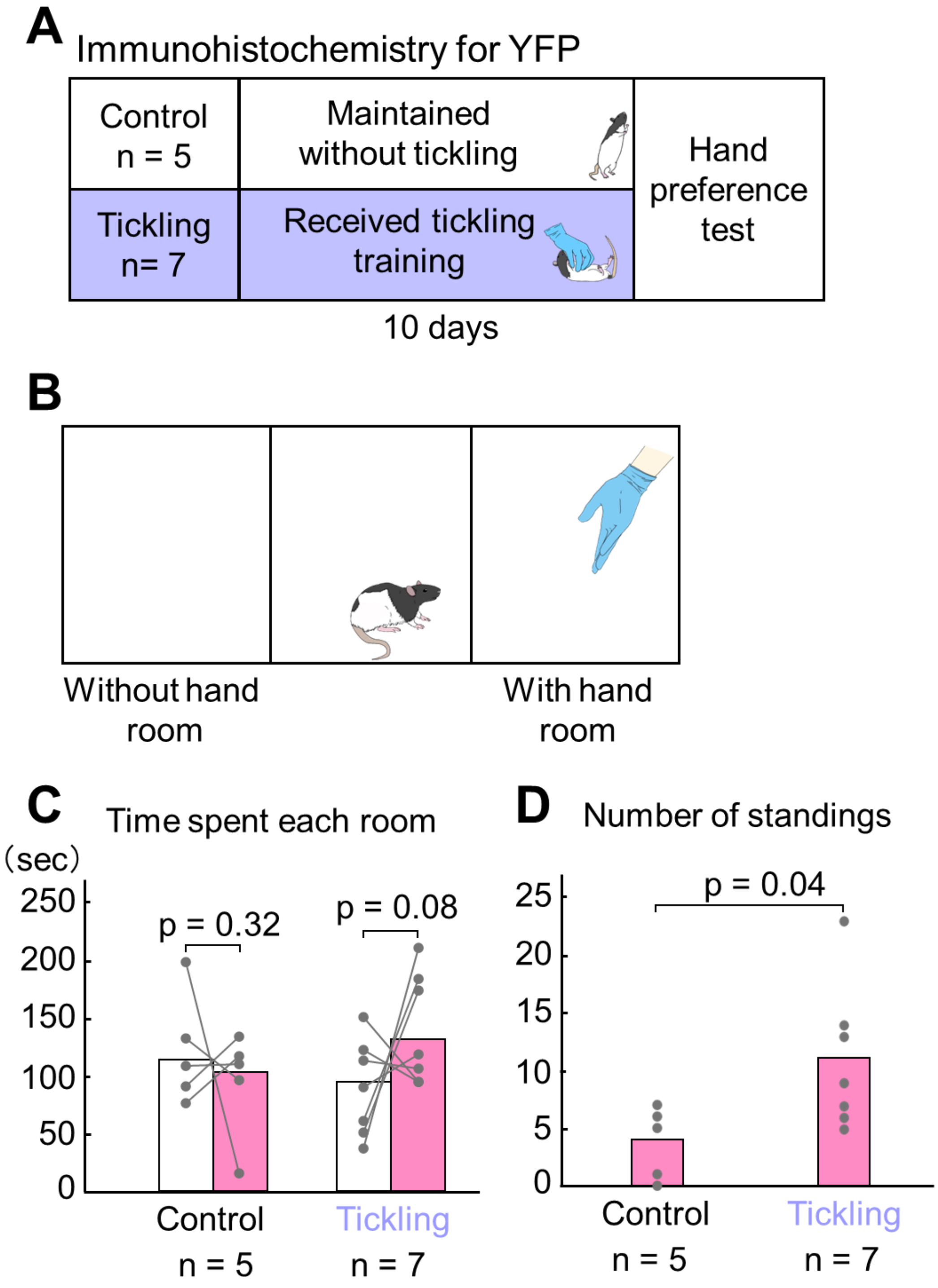

Figure S3. Evidence of Rats Preferring Tickling Over Just a Hand, Related to Figure 2.

(A) To verify that the prefered to the tickling room was specifically driven by tickling, the rats that had received tickling training or maintained without training were conducted

hand preference test. (B) Hand preference test was performed using the same CPP apparatus and consisted of a room presenting in a human hand and a room with nothing. The rats were tested to measure duration in each room and frequency of standing for 5 minutes. (C) Although time spent in each room was not altered regardless of whether received tickling training, time in with hand room was significantly increased in the tickling group compared to the control group. (D) Furthermore, the number of standings in the tickling group significantly increased compared to the control group.

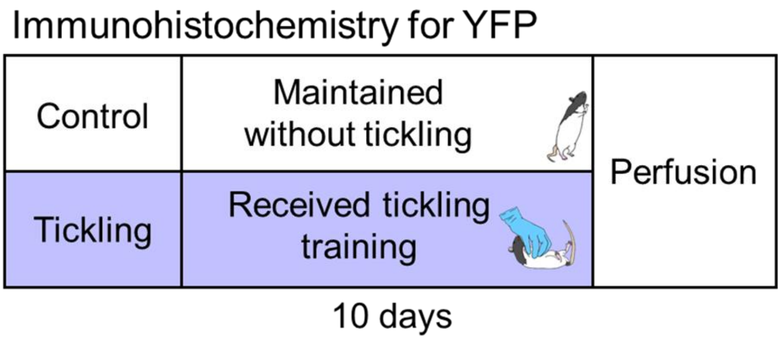

Figure S4. Analysis of Brain Oxytocin Receptor (OTR) Expression Following Tickling-Induced Increased Affinity for Human Hands, Related to Figure 3.

We examined OTR expression levels in brain regions potentially responsive to repeated tickling. The study compared two groups: a control group maintained without tickling training, and an experimental group that underwent daily tickling training for ten days starting at 5 weeks of age.

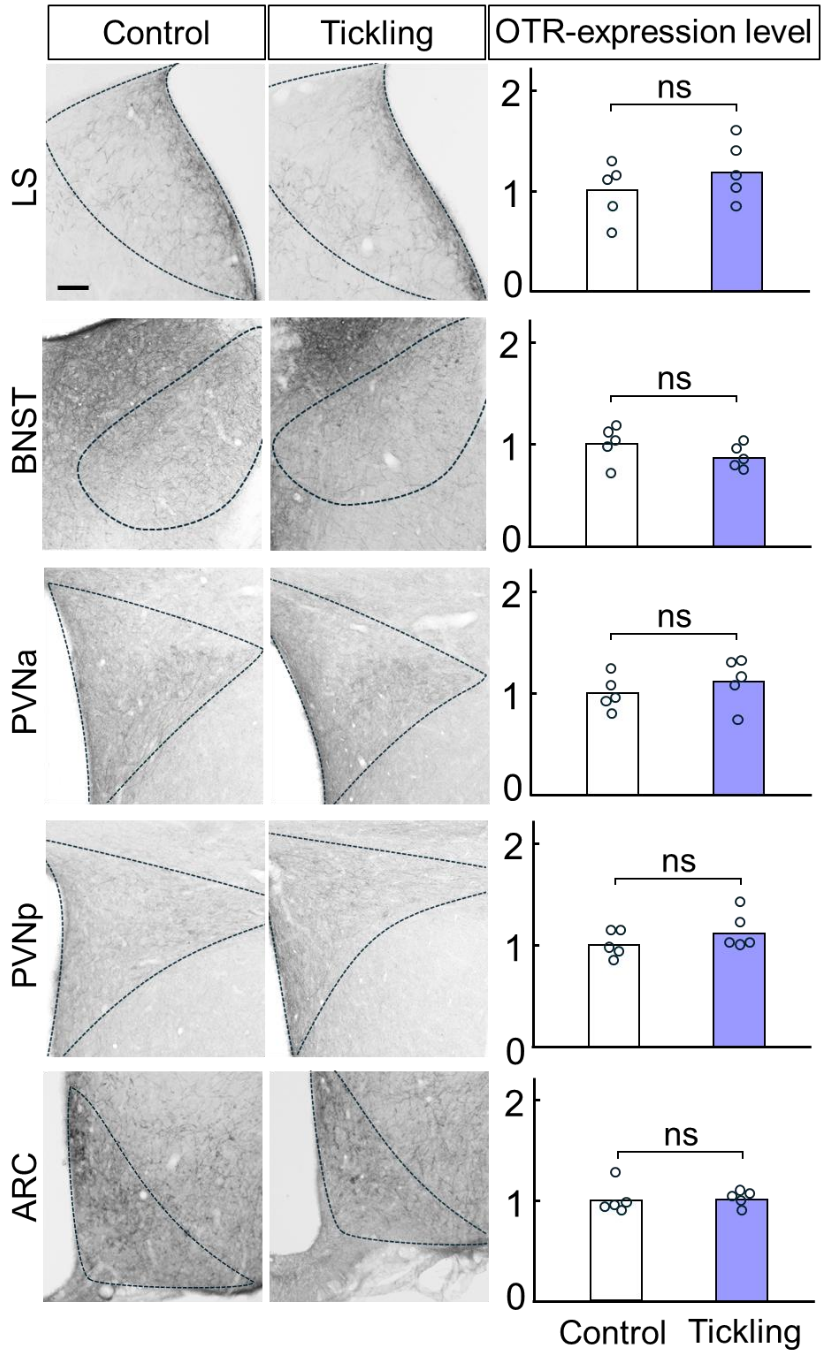

Figure S5. Brain Regions Showing No Changes in Oxytocin Receptor (OTR) Expression Following Tickling Stimulation, Related to Figure 3.

Representative images of GFP-immunostaining showing OTR expression levels. All images were taken at the same magnification. Scale bar = 100 μm. OTR expression

levels were compared between control and tickling groups in the lateral septum (LS), bed nucleus of the stria terminalis (BNST), paraventricular nucleus anterior part (PVNa), paraventricular nucleus posterior part (PVNp), and arcuate nucleus (ARC). No significant differences were observed between groups.

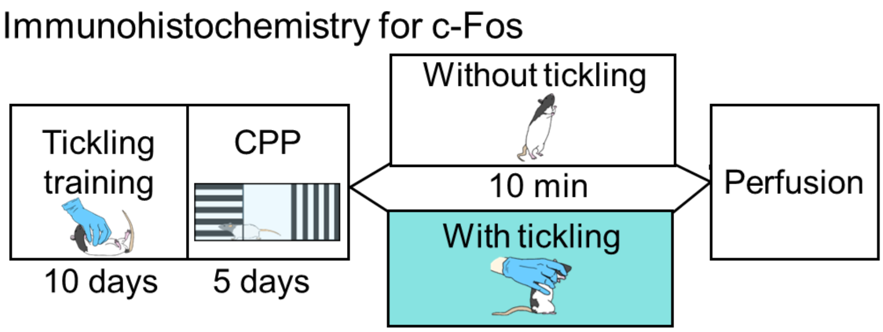

Figure S6. Assessment of Neural Activation in Oxytocin Receptor (OTR)-Enriched Regions Following Tickling, Related to Figure 3.

Neural activation was examined using c-Fos immunoreactivity in brain regions that showed increased OTR expression after tickling. All rats underwent ten days of tickling training and were then divided into two groups: one group received a final tickling session before perfusion, while the other group did not.

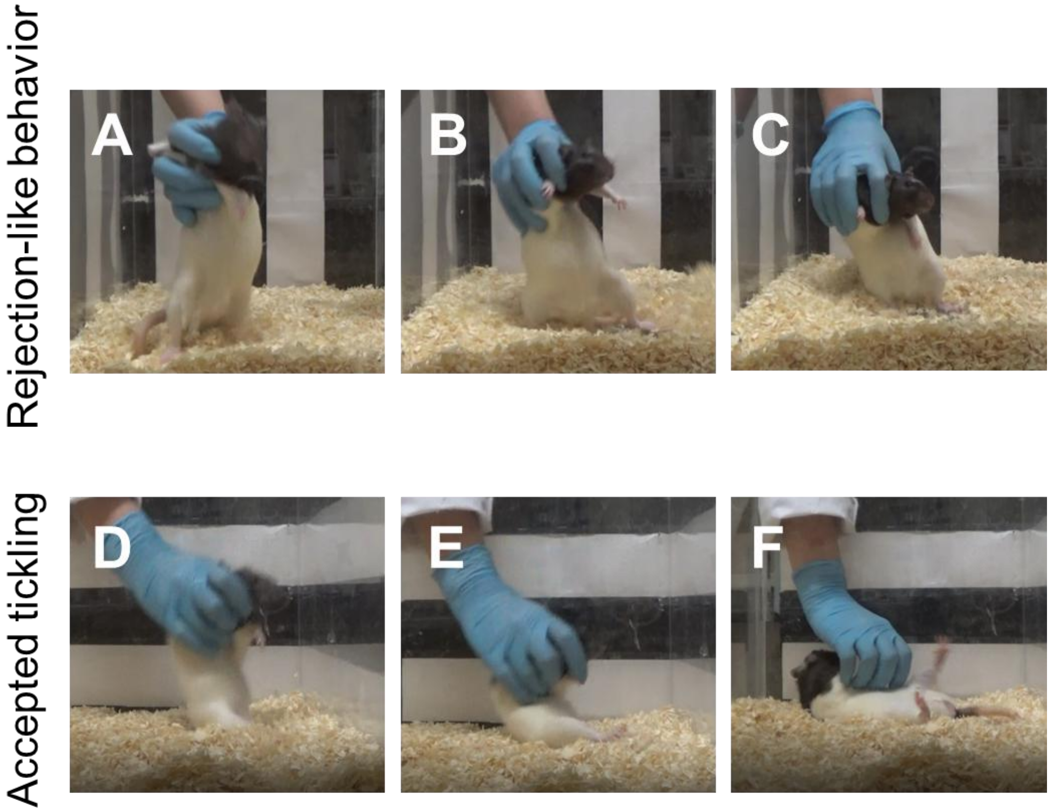

Figure S7. Documentation of Tickling-Rejection-like Behavior, Related to Figure 5, 6, and Video S3.

(A, B, C) Representative photographs showing rats displaying rejection-like behavior during tickling, characterized by bracing their hind legs to resist being rolled over. (D, E, F) In contrast, these photographs showing rats displaying accepted tickling, characterized by their hind legs lifted off the floor and rolling onto their backs.

			Statistical analyzed method		statistical values	Effect size	P value
**Figure 2. Rats developed an increased affinity and formed affinity for human hands through pleasurable tickling.**							
Time in tickling room			two-way ANOVA	interaction	$F_{1,5}$ = 22.16	η² = 0.94	p = 0.01
			Bonferroni post hoc test	Pre vs Post	t = 2.52	d = 0.05	p = 0.03
Number of standings			two-way ANOVA	interaction	$F_{1,5}$ = 9.43	η² = 0.78	p = 0.03
			Bonferroni post hoc test	Pre vs Post	t = 3.56	d = 0.10	p = 0.005
Total time of standings			two-way ANOVA	interaction	$F_{1,5}$ = 19.67	η² = 0.70	p = 0.01
			Bonferroni post hoc test	Pre vs Post	t = 2.49	d = 0.07	p = 0.04
**Figure 3. Repeated pleasant touch tickling increased the expression of oxytocin receptor and c-Fos in the brain.**							
OTR-expression level	NAc		Student's t-test		t_8 = 3.22	d = 2.14	p = 0.02
	VMHvl		Student's t-test		t_8 = 3.21	d = 2.27	p = 0.01
	MePD		Student's t-test		t_8 = 1.59	d = 1.12	p = 0.15
Number of c-Fos$^+$ cells	NAc		Student's t-test		t_{11} = 5.09	d = 3.15	p < 0.001
	VMHvl		Student's t-test		t_{11} = 2.66	d = 1.65	p = 0.02
	MePD		Student's t-test		t_{11} = 1.79	d = 1.11	p = 0.10
**Figure 4. Increase in oxytocin fibers in the ventrolateral part of the ventromedial hypothalamus (VMHvl) following tickling.**							
PS60 VMH			Student's t-test	Control vs Tickling	t_6 = 4.49	d = 3.66	p = 0.004
PS41 VMH			Student's t-test	Control vs Tickling	t_6 = 1.76	d = 1.44	p = 0.13
**Figure 5. Chemogenetic inhibition of oxytocin receptor (OTR) neurons in the ventrolateral part of the ventromedial hypothalamus (VMHvl) prevented affinity-like behaviors from rats to human hands contact that promoted pleasant touch sensation.**							
Time in tickling room	hM4Di (－)	vehicle (1)	two-way ANOVA	interaction	F1,5 = 18.25	η2 = 0.94	p = 0.007
			Bonferroni post hoc test	Pre vs Post	t = 4.74	d = 0.03	p = 0.001
		DCZ	two-way ANOVA	interaction	F1,5 = 134.10	η2 = 0.96	p < 0.001
			Bonferroni post hoc test	Pre vs Post	t = 3.71	d = 0.03	p = 0.01
		vehicle (2)	two-way ANOVA	interaction	F1,5 = 51.92	η2 = 0.97	p < 0.001
			Bonferroni post hoc test	Pre vs Post	t = 4.73	d = 0.06	p = 0.001
	hM4Di (＋)	vehicle (1)	two-way ANOVA	interaction	F1,9 = 36.79	η2 = 0.89	p < 0.001
			Bonferroni post hoc test	Pre vs Post	t = 5.98	d = 0.008	p < 0.001
		DCZ	two-way ANOVA	interaction	F1,9 = 4.09	η2 = 0.87	p = 0.07
			Bonferroni post hoc test	Pre vs Post	t = 3.40	d = 0.03	p = 0.09
		vehicle (2)	two-way ANOVA	interaction	F1,9 = 18.27	η2 = 0.89	p = 0.002
			Bonferroni post hoc test	Pre vs Post	t = 6.79	d = 0.04	p < 0.001
Number of standings	hM4Di (－)	vehicle (1)	two-way ANOVA	interaction	F1,5 = 10.32	η2 = 0.88	p = 0.02
			Bonferroni post hoc test	Pre vs Post	t = 5.76	d = 0.37	p < 0.001
		DCZ	two-way ANOVA	interaction	F1,5 = 9.10	η2 = 0.87	p = 0.03
			Bonferroni post hoc test	Pre vs Post	t = 1.01	d = 0.05	p = 0.34
		vehicle (2)	two-way ANOVA	interaction	F1,5 = 10.22	η2 = 0.81	p = 0.02
			Bonferroni post hoc test	Pre vs Post	t = 2.17	d = 0.11	p = 0.05
	hM4Di (＋)	vehicle (1)	two-way ANOVA	interaction	F1,9 = 8.98	η2 = 0.75	p = 0.02
			Bonferroni post hoc test	Pre vs Post	t = 4.90	d = 0.22	p < 0.001
		DCZ	two-way ANOVA	interaction	F1,9 = 5.21	η2 = 0.77	p = 0.05
			Bonferroni post hoc test	Pre vs Post	t = 1.44	d = 0.05	p = 0.16
		vehicle (2)	two-way ANOVA	interaction	F1,9 = 4.58	η2 = 0.83	p = 0.06
			Bonferroni post hoc test	Pre vs Post	t = 3.01	d = 0.08	p = 0.008
Total time of standings	hM4Di (－)	vehicle (1)	two-way ANOVA	interaction	F1,5 = 12.16	η2 = 0.80	p = 0.02
			Bonferroni post hoc test	Pre vs Post	t = 4.57	d = 0.12	p = 0.001
		DCZ	two-way ANOVA	interaction	F1,5 = 46.93	η2 = 0.88	p = 0.001
			Bonferroni post hoc test	Pre vs Post	t = 2.32	d = 0.05	p = 0.06
		vehicle (2)	two-way ANOVA	interaction	F1,5 = 13.42	η2 = 0.89	p = 0.01
			Bonferroni post hoc test	Pre vs Post	t = 2.04	d = 0.03	p = 0.08
	hM4Di (＋)	vehicle (1)	two-way ANOVA	interaction	F1,9 = 8.49	η2 = 0.82	p = 0.02
			Bonferroni post hoc test	Pre vs Post	t = 5.35	d = 0.07	p < 0.001
		DCZ	two-way ANOVA	interaction	F1,9 = 3.04	η2 = 0.84	p = 0.12
			Bonferroni post hoc test	Pre vs Post	t = 0.34	d = 0.006	p = 0.74
		vehicle (2)	two-way ANOVA	interaction	F1,9 = 14.95	η2 = 0.80	p = 0.004
			Bonferroni post hoc test	Pre vs Post	t = 0.16	d = 0.002	p = 0.88
Number of 50 kHz USV	hM4Di (-) vs hM4Di (+)		two-way ANOVA	interaction	F2,14 = 7.93	η2 = 0.60	p = 0.002
			Bonferroni post hoc test	vehicle (1)	t = 0.86	d = 0.001	p = 0.40
				DCZ	t = 3.74	d = 0.01	p = 0.001
				vehicle (2)	t = 1.88	d = 0.003	p = 0.08
Number of accepted tickling	hM4Di (-) vs hM4Di (+)		two-way ANOVA	interaction	F2,14 = 8.0	η2 = 0.71	p = 0.002
			Bonferroni post hoc test	vehicle (1)	t = 0.86	d = 0.09	p = 0.10
				DCZ	t = 4.33	d = 0.34	p < 0.001
				vehicle (2)	t = 1.74	d = 0.15	p = 0.09
Total time of accepted tickling	hM4Di (-) vs hM4Di (+)		two-way ANOVA	interaction	F2,14 = 13.20	η2 = 0.72	p < 0.001
			Bonferroni post hoc test	vehicle (1)	t = 1.48	d = 0.06	p = 0.15
				DCZ	t = 6.88	d = 0.31	p < 0.001
				vehicle (2)	t = 0.86	d = 0.02	p = 0.40
Rate in accepted tickling	hM4Di (-) vs hM4Di (+)		two-way ANOVA	interaction	F2,14 = 19.84	η2 = 0.82	p < 0.001
			Bonferroni post hoc test	vehicle (1)	t = 1.85	d = 0.09	p = 0.07
				DCZ	t = 9.74	d = 0.14	p < 0.001
				vehicle (2)	t = 0.44	d = 0.05	p = 0.67
Time spent toward human hand	hM4Di (-) vs hM4Di (+)		two-way ANOVA	interaction	F2,14 = 15.35	η2 = 0.77	p < 0.001
			Bonferroni post hoc test	vehicle (1)	t = 5.37	d = 0.06	p = 0.004
				DCZ	t = 5.48	d = 0.10	p < 0.001
				vehicle (2)	t = 1.40	d = 0.02	p = 0.17

**Figure 6. Oxytocin receptor (OTR) antagonism in the ventrolateral part of the ventromedial hypothalamus (VMHvl) prevented affinity-like behaviors from rats to human hands promoted by pleasant touch sensation.**						
Time in tickling room	vehicle (1)	two-way ANOVA	interaction	F1,6 = 14.51	η2 = 0.92	p = 0.009
		Bonferroni post hoc test	Pre vs Post	t = 2.47	d = 0.07	p = 0.04
	OTA	two-way ANOVA	interaction	F1,6 = 2.63	η2 = 0.80	p = 0.16
		Bonferroni post hoc test	Pre vs Post	t = 1.17	d = 0.008	p = 0.26
	vehicle (2)	two-way ANOVA	interaction	F1,6 = 16.07	η2 = 0.91	p = 0.007
		Bonferroni post hoc test	Pre vs Post	t = 3.52	d = 0.03	p = 0.005
Number of standings	vehicle (1)	two-way ANOVA	interaction	F1,6 = 6.83	η2 = 0.83	p = 0.04
		Bonferroni post hoc test	Pre vs Post	t = 3.11	d = 0.35	p = 0.009
	OTA	two-way ANOVA	interaction	F1,6 = 3.21	η2 = 0.87	p = 0.12
		Bonferroni post hoc test	Pre vs Post	t = 3.90	d = 0.51	p = 0.003
	vehicle (2)	two-way ANOVA	interaction	F1,6 = 22.20	η2 = 0.79	p = 0.003
		Bonferroni post hoc test	Pre vs Post	t = 9.04	d = 0.55	p < 0.001
Total time of standings	vehicle (1)	two-way ANOVA	interaction	F1,6 = 7.42	η2 = 0.69	p = 0.04
		Bonferroni post hoc test	Pre vs Post	t = 6.47	d = 0.26	p < 0.001
	OTA	two-way ANOVA	interaction	F1,6 = 2.62	η2 = 0.79	p = 0.16
		Bonferroni post hoc test	Pre vs Post	t = 4.11	d = 0.16	p = 0.003
	vehicle (2)	two-way ANOVA	interaction	F1,6 = 6.53	η2 = 0.80	p = 0.04
		Bonferroni post hoc test	Pre vs Post	t = 6.90	d = 0.03	p < 0.001
Number of 50 kHz USV		one-way ANOVA	main effect	F2,6 = 9.04	η2 = 0.81	p = 0.004
		Bonferroni post hoc test	vehicle (1) vs OTA	t = 4.17	d = 0.02	p = 0.004
			vehicle (2) vs OTA	t = 2.80	d = 0.01	p = 0.04
			vahicle (1) vs vehicle (2)	t = 1.37	d = 0.004	p = 0.58
Number of accepted tickling		one-way ANOVA	main effect	F2,6 = 22.01	η2 = 0.76	p < 0.001
		Bonferroni post hoc test	vehicle (1) vs OTA	t = 6.34	d = 0.52	p < 0.001
			vehicle (2) vs OTA	t = 4.87	d = 0.96	p = 0.001
			vahicle (1) vs vehicle (2)	t = 1.47	d = 0.10	p = 0.50
Total time of accepted tickling		one-way ANOVA	main effect	F2,6 = 11.51	η2 = 0.57	p = 0.002
		Bonferroni post hoc test	vehicle (1) vs OTA	t = 4.54	d = 0.04	p = 0.002
			vehicle (2) vs OTA	t = 3.61	d = 0.03	p = 0.01
			vahicle (1) vs vehicle (2)	t = 0.93	d = 0.005	p = 1.0
Rate in accepted tickling		one-way ANOVA	main effect	F2,6 = 43.76	η2 = 0.88	p < 0.001
		Bonferroni post hoc test	vehicle (1) vs OTA	t = 5.99	d = 0.11	p < 0.001
			vehicle (2) vs OTA	t = 9.22	d = 0.23	p < 0.001
			vahicle (1) vs vehicle (2)	t = 3.23	d = 0.21	p = 0.02
Time spent toward human hand		one-way ANOVA	main effect	F2,6 = 57.68	η2 = 0.79	p < 0.001
		Bonferroni post hoc test	vehicle (1) vs OTA	t = 9.65	d = 0.13	p < 0.001
			vehicle (2) vs OTA	t = 8.91	d = 0.17	p < 0.001
			vahicle (1) vs vehicle (2)	t = 0.75	d = 0.01	p = 1.0
**Figure S2. Behavioral changes in the non-tickling room following increased affinity for human hands through pleasurable tickling.**						
Time in non-tickling room		two-way ANOVA	interaction	$F_{1,5}$ = 22.16	η^2 = 0.94	p = 0.01
		Bonferroni post hoc test	Pre vs Post	t = 5.82	d = 0.06	p < 0.001
Number of standings		two-way ANOVA	interaction	$F_{1,5}$ = 9.43	η^2 = 0.78	p = 0.03
		Bonferroni post hoc test	Pre vs Post	t = 2.13	d = 0.09	p = 0.05
Total time of standings		two-way ANOVA	interaction	$F_{1,5}$ = 19.67	η^2 = 0.70	p = 0.01
		Bonferroni post hoc test	Pre vs Post	t = 1.97	d = 0.03	p = 0.09
**Figure S3. Evidence of rats prefer tickling over just a hand.**						
Time spent each room		two-way ANOVA	interaction	$F_{1,10}$ = 2.15	η^2 = 0.98	p = 0.17
	control group	Bonferroni post hoc test	without hand room vs with hand	t = 1.04	d = 0.007	p = 0.32
	tickling group		without hand room vs with hand	t = 1.93	d = 0.008	p = 0.08
	without hand room		control group vs tickling group	t = 1.68	d = 0.007	p = 0.12
	with hand room		control group vs tickling group	t = 2.40	d = 0.006	p = 0.03
Number of standings		Student's t-test	control group vs tickling group	t_{10} = 2.34	d = 1.50	p = 0.04
**Figure S5. Brain regions showing no changes in oxytocin receptor (OTR) expression following tickling stimulation.**						
LS		Student's t-test	Control vs Tickling	t_8 = 1.09	d = 0.77	p = 0.31
BNST		Student's t-test	Control vs Tickling	t_8 = 1.32	d = 0.93	p = 0.22
PVNa		Student's t-test	Control vs Tickling	t_8 = 0.95	d = 0.67	p = 0.37
PVNp		Student's t-test	Control vs Tickling	t_8 = 1.20	d = 0.85	p = 0.26
ARC		Student's t-test	Control vs Tickling	t_8 = 0.15	d = 0.10	p = 0.88

Table S1. Summary of Statistical Analysis of Experimental Data, Related Figure 2, 4, 5, and 6.
